# Gleanings

**Published:** 1894-05

**Authors:** F. H. Lutze

**Affiliations:** Brooklyn, N. Y.


### GLEANINGS.

F. H. LUTZE, M. D., BROOKLYN, N. Y.

Sciatica, heavy, stiff cramp in calves at night; in soles at every step, burning of soles. Sulph.

— lassitude, startings at night awaken him; pains in calves all night; chorea-like motion, jerking, trembling. Argent-nitr.

— rending, shooting, worse from walking P. M., and at night, better from pressure; restless, sleepless at night. Coffea.

— from working in water. Calc-c.

— pain in right leg, only when moving or sitting up, pain shooting the whole length of the leg, better lying perfectly still. Diosc.

— burning, shooting down to the left foot, greatly aggravated from moderate motion, better rapid motion. Iris-vers.

— chronic, pain worse three to five A. M. Sep.

— pain in sacrum, down right thigh, worse lying on affected side, lying down at night, straining at stool, coughing, sneezing, worse right side. Tellur.

— intense pains, extending to the ramifications of the nerve, with feeling of numbness, which sometimes alternates with the pains; pains extend into toes, anterior crural neuralgia. Gnaphal.

— pains sharp, transient, darting upward and downward, from both sides to centre, worse from heat, better from cold, attacks with cold perspiration. Veratr-alb.

### SLEEP AND DREAMS.

Awaking at two A. M. Ammon-m., Lachnant.

— — two to three A. M., can't sleep again. Magnes-c.

— — two to four with all ailments. Kali-c.

— — three A. M. Baptis., Borax, Chin., Dulcam.; worst sleep after three A. M.

— — three A. M. and falls asleep again. } Sepia.

or cannot sleep again. }

Awaking at three A. M. and falls asleep again late mornings. Zingiber.

Sleepless before twelve P. M. Bry., Calc-phos., Chelidon., Chin.

— worse after three A. M. Dulc., Con., Creosote, Lil-tig. (Merc-iod. until one A. M.), Mur-ac., Op., Phos., restless, Puls., Rhus-t., Selen., Valerian.

— after twelve P. M. Acon., Asafœt., Aur., Euphras., Ferr., Helon., Iod., Merc-iod-rub., Mezer., Nitr., Psor., Ran-scel., Rhod., Sabin.

Restless after three A. M.; sleeps till three A. M. Baptis.

Sleep disturbed by very light noise. Caladium.

— — even by rattling of paper. Caladium.

Hungry at night, must eat after twelve P. M., with suffocative spells. Graph.

— — — on awaking. Lycopod.

— — midnight. Psorin.

— — — canine hunger. Phos-ac., Carb-v., Kali-iod., Selen., Staph.

Awakens hungry. Phos., Chin., Teucrium.

— — must eat bread at midnight. Psor.

Bed feels too hard. Arn., Baptis.

Sleeplessness of business men and students, a wide-awake feeling; *not a wink of sleep last night;* also talking in sleep. Gels., Coffea, Op.

— from gastric causes. Ars.

— — chronic — — with gas. Nux-v., China.

— — enteric causes. Bell., Cham., Lycopod., Sulph.

Sleeplessness from pulmonary causes. Bell., Bry., Veratr-vir., Phos.

— from melancholia. Cimicif.

— — mental irritability with tendency to delirium. Hyos.

— — excitement. Coffea.

— after exhaustion or fevers. Mosch.

Babies cry all day and sleep soundly all night. Lycopod.

— when sick will not sleep day or night, but worry, fret, and cry. Psor.

Babies are good or rest all day and raise Cain all night. Jalap.

— fall asleep, sleep from ten to thirty seconds, then wake with a start and scream. Lachesis.

— cries all night, about daylight goes to sleep and sleeps all the forenoon. Calc-c. (Syphilin).

Sleepy during and after eating. Bovista, Kali-c., Phos., Puls.

Sleepless in early part of night. Valer.

### Fever, Chill, Perspiration.

Chill begins in elbows and knees. Natr-m.

— from wet cold, especially on drinking cold liquids when overheated. Bellis-peren.

Gooseflesh when warm. Puls.

In intermittents, patient wants to be held during chill. Gels.

— — — — some one to lie on him; or to be held close. Lachesis.

Chill with hunger at the outset, during chill, soles of feet become cold. Phytolacca.

Flushes of heat at climacteric. Ambr-gris., Cimicif., Coccul., Lachesis, Lycopod., Puls., Sang., Sep., Veratr-vir., Sulph., Oleum-an. at 1.30 P. M.

Perspiration cold on forehead. Acetic-ac., Acon., Asafœt., Benz-ac., Gels., Graph., Lachesis, Nux-v., Puls., on one side; Veratr-alb. comes out cold.

— on uncovered parts. Thuja.

Sweat, wants to be covered during sweat. Æthusa.

Covers up during fever and pains in abdomen; when the skin becomes cold he uncovers; vomiting and purging with cold, blue, dry skin. Camph.

Chills severe shaking, with scarcely any coldness, vomit after each drink of water, vomit bile at the close of the hot stage, followed by a little perspiration. Eupator-perf.

— over back worse in the evening, as if dashed with cold water, or like cold water coursing through veins, restlessness, tearing pains all through the body, delirium. Rhus-tox.

Perspiration cold on lumbar and sacral region worse during stool. Kali-bichro., Sulph., Plantago.

### Skin.

Pain in an old scar of an old abscess. Calc-phos.

Ecchymoses remaining a long time after bruises. Ledum.[30]; internally.

Ulcers; become deep without spreading. Kali-bichro.

— deep, especially chancres with indurated edges. Nitr-acid.

Small-pox with purging. Potentilla-torment.

Warts on palms. Anacard.

— smooth. Anti-crud.

— solid with horny tops. Caust.

— fissured and cut up, with an appearance of cauliflower. Thuja.

Ecchymoses blue. Nux-vom.

— bluish red. Bell.

— blue spots on skin. Ars., Op.

Skin pale blue. Plumb.

Abscess; slight pain; swelling with pale red blush; hard and heavy. Bry.

Ulcers very red and fiery looking; on legs; in mouth; throat; nodes on shin-bones; very angry looking chancres. Cinnabar.

Ulcer, red flat on glans and inner surface of prepuce; secreting yellow ichor. Corall-rub.

Hide-bound, sensation of. Crot-tig.

Eczema oozing a sticky glutinous fluid, or transparent watery. Graph.

Ulcers like a lump of lard with a hole in it; cancerous ulcers; great sensitiveness to slightest touch, even of clothes. Hep-s-c.

— become deep, without spreading, look as if cut out with a punch. Kali-bichro.

Ecchymoses remaining a long time in bruises or contused parts, after the pains and inflammation have subsided. Ledum-pal.

Skin chapping, want of perspiration. Oleand.

Erysipelas of scalp; left side of face going to right. Rhus-t.

Blisters spreading with a red edge in advance (black edge, Ars.); phlegmonous, especially if it begins in ankles and runs gradually up the leg into deeper tissues; no fever. Rhus-t.

Itching in various parts worse in legs while undressing; cannot wear flannels; worse from any change of temperature. Rumex (Graph. and Caust. follow well after Rumex).

Chapping or cracking of skin may be deep, worse from washing in water. Sep., Calc-c.

Eruptions do not come out; crying out in a frightened manner, as soon as patient falls asleep. Stram.

